# Electromechanical Cornea Reshaping for Refractive Vision Therapy

**DOI:** 10.1021/acsbiomaterials.2c01177

**Published:** 2023-01-12

**Authors:** Anna M. Stokolosa, Jack Thomas-Colwell, Katelyn K. Dilley, Yueqiao Qu, Charlotte Cullip, Andrew E. Heidari, Michelle Huang, Nathalie Kerrigan, Kellie Hsu, Jack Leonard, Karthik R. Prasad, Brian J.F. Wong, Michael G. Hill

**Affiliations:** † Department of Chemistry, Occidental College, Los Angeles, California 90041, United States; ‡ Beckman Laser Institute & Medical Clinic, University of California, Irvine, Irvine, California 92697, United States; § Department of Biomedical Engineering, University of California, Irvine, Irvine, California 92697, United States; ∥ Department of Otolaryngology-Head and Neck Surgery, School of Medicine, University of California, Irvine, Orange, California 92617, United States

**Keywords:** electrochemistry, stress-relaxation, cornea, vision refraction, optical coherence tomography (OCT)

## Abstract

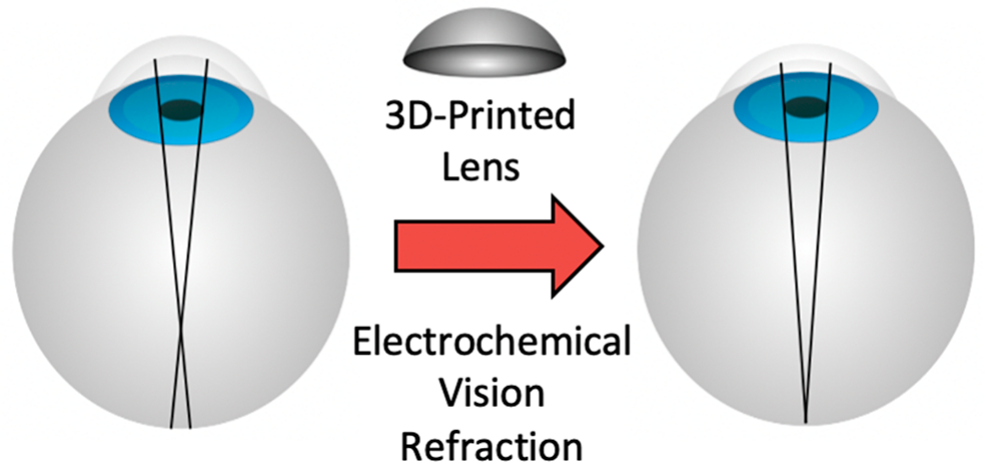

The corneal stroma consists of orthogonally stacked collagen-fibril lamellae that determine the shape of the cornea and provide most of the refractive power of the eye. We have applied electromechanical reshaping (EMR), an electrochemical platform for remodeling cartilage and other semirigid tissues, to change the curvature of the cornea as a potential procedure for nonsurgical vision correction. EMR relies on short electrochemical pulses to electrolyze water, with subsequent diffusion of protons into the extracellular matrix of collagenous tissues; protonation of immobilized anions within this matrix disrupts the ionic-bonding network, leaving the tissue transiently responsive to mechanical remodeling. Re-equilibration to physiological pH restores the ionic matrix, resulting in persistent shape change of the tissue. Using *ex vivo* rabbit eyes, we demonstrate here the controlled change of corneal curvature over a wide range of refractive powers with no loss of optical transparency. Optical coherence tomography (OCT), combined with second-harmonic generation (SHG) and confocal microscopy, establish that EMR enables extremely fine control of corneal contouring while maintaining the underlying macromolecular collagen structure and stromal cellular viability, positioning electrochemical vision therapy as a potentially simple and ultralow-cost modality for correcting routine refractive errors.

## Introduction

The cornea is a transparent, highly organized anatomical structure that provides most of the refractive power of the eye. Natural variation, birth defects, trauma, and various pathologies can alter the shape, structural stability, and transparency of the cornea, leading to impaired vision.^1,2^ For example, in the United States, myopia and high myopia (spherical equivalent refractive errors of ≤−0.5 and ≤−6.0 diopters, respectively) affect over 40% of the population. Surgical intervention to treat common refractive errors (i.e., myopia, hyperopia, and astigmatism) typically involves recontouring the corneal curvature by physically ablating corneal tissue, e.g., laser-assisted in situ keratomileusis (LASIK) and photorefractive keratectomy (PRK). Significantly, both of these procedures permanently reduce the biomechanical strength of the cornea, raising the potential risk of post-treatment ectasias resulting from a weakened corneal structure. Other possible side effects include excessive glare and “halos” associated with diminished night-vision acuity.^3,4^ Nonsurgical therapies also have significant downsides. For example, orthokeratology temporarily changes the refractive power of the eye by molding the corneal surface via hard contact lenses worn at night. Drawbacks of this strategy include a long treatment period (typically weeks) to achieve near-emmetropic acuity; the requirement for nightly installation of “retainer” lenses to prevent shape recidivism; and elevated risks of bacterial, protozoan, and herpectic keratitis.

Here we report an alternative, molecular-based approach to vision refraction that relies instead on transiently altering the chemical properties and equilibrium stress distribution of the cornea via electrochemically generated pH gradients within the corneal tissue. Using fresh *ex vivo* New Zealand rabbit globes as test subjects, electromechanical reshaping (EMR), a noninvasive, inexpensive surgical technique developed originally for reshaping cartilage and other collagen rich tissues,^5−8^ allows for the controlled change of corneal curvature over a wide range of refractive powers, with no loss of transparency or apparent damage to the underlying tissue.

## Methods

*Ex vivo* eye specimens from 10–12-week-old New Zealand White rabbits were obtained from a local abattoir. After enucleation, globes were stored in sterile phosphate-buffered saline (PBS), pH 7.4, and were treated within 24 h. Between 8 and 10 eyes were evaluated for shape change, cellular viability, and stromal organization, depending upon the imaging method used.

Custom reshaping lenses were prepared by press molding 25 μm thick platinum-foil disks (8 mm diameter) onto 3D-printed hemispheres (FormLabs Form 3 Stereolithographic printer) of varying focal lengths. After fastening a strip of copper tape to the press-molded platinum, a 3D-printed ring was placed around the hemisphere and filled with epoxy resin. Upon curing, the ring was removed from the mold to give an EMR reshaping contact lens that featured a concave platinum-electrode surface of specified curvature. The reference electrode was incorporated into the lens by including a raised detail that encircled the mold, producing a channel in the contact lens into which a silver-wire pseudoreference electrode was threaded. Electrodes were connected to a potentiostat through apertures on opposite sides of the support ring. “Half-moon” lenses were designed with one hemisphere of the platinum surface being electroactive with copper tape attached to one edge of the press-molded platinum. A nonconductive hemisphere was electrically isolated by scoring the platinum electrode in half with a razor blade after epoxy cure. Images and metrics for the lenses are provided in the Supporting Information.

### Electrochemistry

Electrochemical treatment was carried out using a CH-Instruments Model 650 Potentiostat/Galvanostat. Prior to treatment, cyclic voltammograms were run in phosphate buffered saline (PBS) using the concave spherical platinum lens as the working electrode, a silver wire pseudo reference electrode, and a platinum wire auxiliary electrode. Eye specimens were mounted into a customized holder and submerged in PBS. EMR involved either (i) bulk electrolysis at a constant 2.0 V vs AgCl/Ag; (ii) cycling the potential (1.5 V vs Ag wire) on-and off at 0.5 Hz; or (iii) looped double-potential-step chronocoulometry between 1.5 V and −0.1 V (vs Ag wire) at 0.5 Hz until the total anodic charge passed was 150 mC (or 75 mC for half-moon lenses), typically ∼2 min.

### Imaging

Three-dimensional spectral domain optical coherence tomography, SD-OCT, imaging was performed (lateral resolution of 11.78 μm and axial resolution of 3.5 μm).^9^ Second-harmonic generation, SHG, images of pre- and post-treatment cornea were captured by mounting the intact globe into a custom jig (see the Supporting Information) such that the cornea made contact with a glass coverslip. The assembly was then positioned onto the viewing platform of a Leica TCS SP8MP8Microscope with a 10× objective, and images were obtained using an excitation wavelength of 810 nm and an emission range of 395–415 nm. SHG z-stacks (900 μm × 900 μm), taken with an average thickness of 150 μm with a 10- μm step, were processed using FIJI open-source imaging software.^10^ After treatment and SD-OCT and SHG imaging, cornea were excised with a corneal trephine and incubated in freshly prepared 4 μM ethidium homodimer-1 (EthD-1)/0.5 μM calcein AM in PBS (1) for 30 min in darkness at room temperature, according to a commercial LIVE/DEAD cell-viability assay (Molecular Probes). After incubation, cornea were rinsed with PBS and mounted onto a custom imaging slide featuring a 3 mm light aperture (Supporting Information). Laser-scanning confocal microscopy was then performed using a Zeiss LSM 980 inverted confocal instrument and viewed through a 10x objective. A tile region was selected with 25 z-slices set to image the entire depth of the cornea, approximately 700 μm. A 488 nm laser was used to excite the calcein AM dye, with emission detected between 489 and 560 nm. A 514 nm laser was used for EthD-1 excitation, with emission detected between 587 and 693 nm. Images were processed and analyzed using FIJI open-source software. For live/dead counting, channels were split and independently converted to binary masks using Otsu Thresholding. After applying these masks to the original images, the inverse of the dead channel was used to mask the live channel as to make the two disjoint. This simulates the worst-possible case, where any overlap is assumed to be dead. The channels were then recombined, and the slices were compiled to give a 3D render. The percent of dead cells for a given slice was gathered by summing the pixel data for both the live and dead channels. These sums were calculated using Fiji’s “Plot Z-axs profile” feature.

## Results and Discussion

The cornea is a layered structure comprised of the epithelium, Bowman’s membrane, stroma, Descemet’s membrane, and the endothelium. The stroma is the largest of these layers and provides most of the structure for maintaining corneal shape;^11^ collagen molecules within the stroma are organized into uniform fibrils that span the entire plane of the structure. Transparency of corneal tissue relies on the precise lattice arrangement of these fibrils to eliminate backscattered light.^12^ In the stroma, Type I collagen triple helices are arranged into orthogonal lamellae;^13^ the individual fibrils have a smaller diameter than in other connective tissues and the overall structure is supported by proteoglycans and Type V collagen. The central region around the apex of the cornea is quasi-spherical, while the periphery adopts a prolate ellipsoidal shape.^14^

From a molecular point of view, the collagen and proteoglycan milieu of the stroma comprise a highly organized polymer hydrogel. Sulfated glycosaminoglycans (GAGs), deprotonated under physiological conditions, provide a substantial fixed negative charge to the tissue, resulting in an ionic-bond network that provides structural rigidity.^15^ Stromal keratocytes that govern homeostasis and repair processes are sparsely populated within this extracellular matrix;^16^ maintaining their viability is paramount to prevent scarring and loss of corneal clarity during regeneration or repair.

In prior mechanistic work focused on hyaline cartilage, we established that EMR relies on electrochemical oxidation of water to dioxygen and protons, with subsequent diffusion of H^+^ into the extracellular matrix.^17^ At a pH threshold of ∼2.5, protonation of immobilized anions within this matrix neutralizes the ionic-bonding network, leaving the tissue transiently responsive to mechanical remodeling. Subsequent re-equilibration to physiological pH restores the ionic matrix, locking in the new form factor of the tissue.

**Figure 1:**
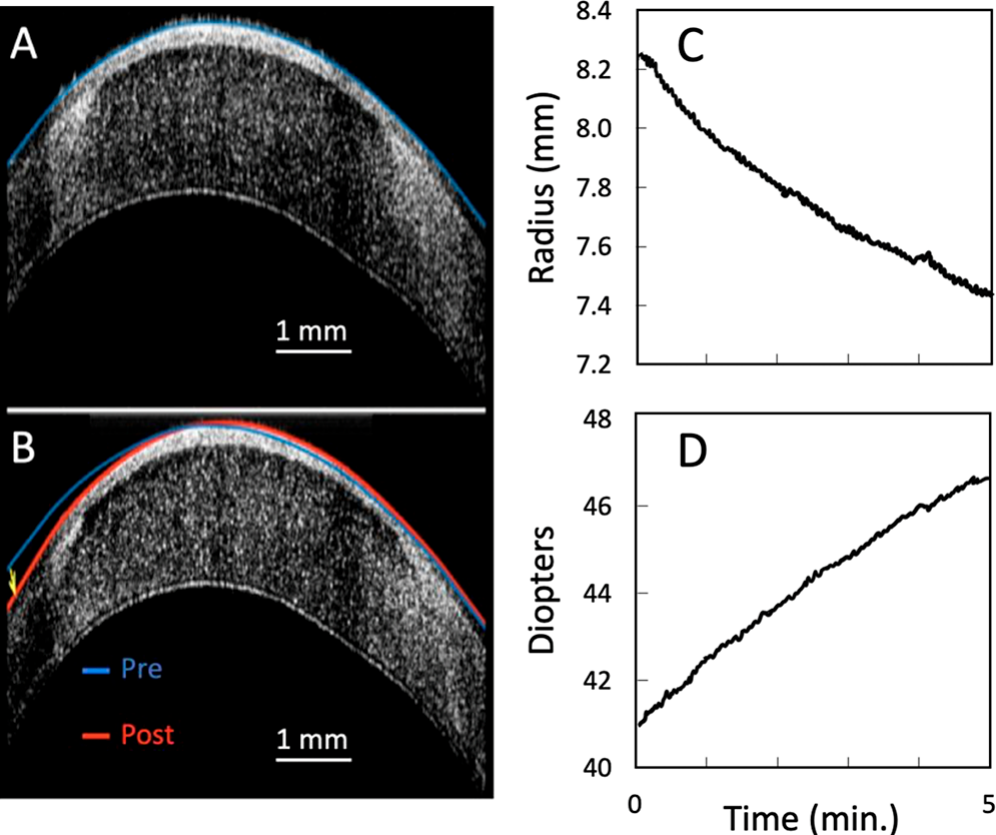
SD-OCT cross section view of cornea (A) pre- and (B) post-treatment using an annular 8 mm platinum ring electrode held at a constant potential of 2 V vs AgCl/Cl for 5 min. Blue and red lines are best fits of the corneal radius of curvature pre- and post-treatment, respectively. Plots of the (C) resulting change in corneal focal length and (D) corresponding change in refractive power are shown at the right.

To test whether EMR could be applied to recontour cornea, we carried out an initial experiment that involved a simple constant-potential treatment. A 50 μm platinum-ring electrode (diameter ∼8 mm) was placed about the apex of the cornea of an *ex vivo* rabbit eye, with platinum auxiliary and AgCl/Ag reference electrodes inserted into a saline solution surrounding the eye globe. Applying a potential of 2 V^18^ resulted in a steady decrease in the focal length of the cornea, as revealed by real-time SD-OCT. Figure 1 shows an SD-OCT cross-section of the anterior chamber (A) before and (B) after treatment, along with the change in (C) focal length (*r*, in mm) and (D) refractive power (in diopters, D) as a function of electrolysis time. Although this treatment left a small opaque region beneath the anode placement, likely due to a combination of dehydration/oxidative damage, the experiment established the possibility of controlling cornea curvature using EMR.

**Figure 2:**
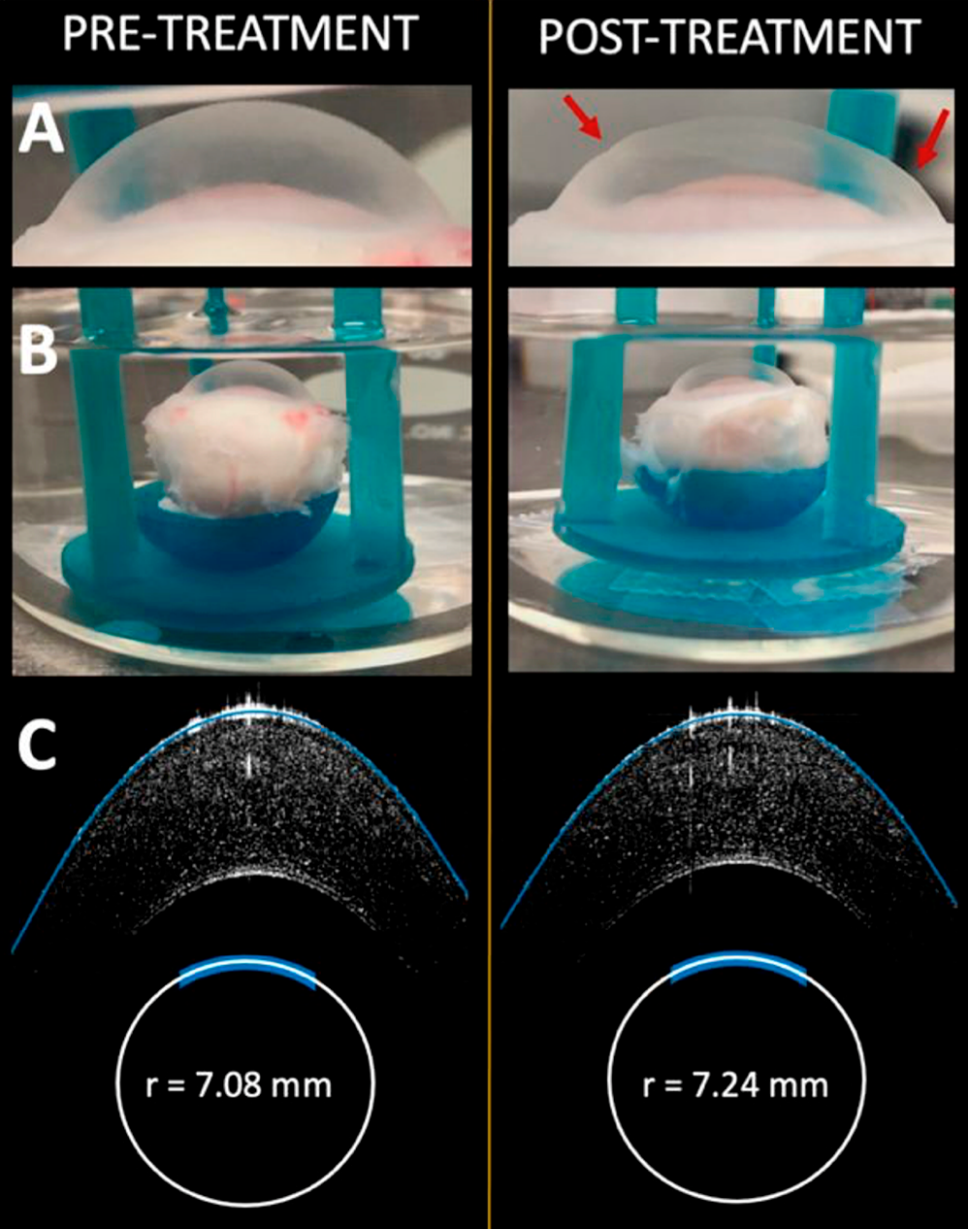
Photographs of rabbit globe in EMR treatment apparatus pre- and post-treatment. (A) The boundary lines of electrode/cornea contact are highlighted in red in the detail image of the post-treatment cornea. (B) Images of globe pre- and post-treatment in PBS buffer. (C) SD-OCT images of the cornea pre- and post-treatment using a 7.25 mm reshaping lens. The radius of curvature is shown below each image, representing a −3.12 diopter change in the refractive power of the cornea.

In order to better achieve refractive correction and to maintain finer control over the shape of the corneal surface, we prepared a custom 3D-printed reshaping lens/eye mount apparatus. Press molded lenses of different refractive power for EMR were assembled by stamping 3D-printed semispherical molds of specified radius of curvature (e.g., focal length) onto 25 μm thick platinum disks 8 mm in diameter. A silver ring served as the pseudoreference electrode. For EMR, excised rabbit eyes were mounted into the reshaping apparatus, cornea facing up (Figure 2). The reshaping lens was then lowered onto the cornea using guide rails to orient the center of the working electrode onto the corneal apex. The apparatus was submerged in phosphate-buffered saline, PBS, pH 7.4, and an auxiliary electrode was placed into the solution.

At our treatment potential, 1.5 V vs the pseudoreference silver electrode, cyclic voltammetry in PBS reveals an anodic current density of ∼2 mA/cm^2^ for the 4e^–^ oxidation of water. Notably, the cyclic voltammograms reveal an additional irreversible feature on the leading edge of this current due to the oxidation of chloride, with subsequent generation of ClO^–^; the return wave due to the reduction of hypochlorite occurs at ∼0.1 V. Although higher applied overpotentials result in larger currents, we carried out the reshaping treatment at as low a potential as possible in an attempt to avoid the electrochemical generation of higher-potential reactive oxygen species such as hydrogen peroxide.

To calculate the appropriate electrochemical “dose” for EMR, we assumed a fixed-charge density of anions within the proteoglycan matrix of the stroma of ∼0.15 M, by analogy to the charge density found in cartilage.^19,20^ Given a 0.5 mm deep treatment “cylinder” (the average depth of the stroma), and the one-to-one correspondence between H^+^ generated and electrons passed during water oxidation, we estimate that an EMR dose between 0.1 and 0.2 C would be required to neutralize most of the fixed-negative charge within the treatment volume. In order to avoid extreme drops in the pH at the corneal surface, the potential was pulsed on and off at 0.5 Hz, targeting a total charge passed of 0.15 C.

Figure 2 shows pre- and post-treatment images of an eye after EMR. In this experiment, a reshaping lens with a 7.25 mm radius of curvature was used on a rabbit eye with an initial focal length of 7.08 mm. The post-treatment photograph clearly shows distinct flattening of the corneal surface; edge effects created at the margins of the lens are clearly visible in the photograph where the corneal surface was in direct contact with the reshaping lens. Significantly, control experiments in which the reshaping lens was applied to the corneal surface but no electrochemical current was passed yielded no changes in the curvature.

Corneal focal lengths were measured both pre- and post-treatment using SD-OCT: as evident in Figure 2C, the curvature of the treated cornea is nearly identical to the spherical radius of the press-molded reshaping lens. Moreover, the post-treated cornea exhibited no discernible loss of transparency, and all of the anatomical features of the eyes (save for their curvatures) appeared to be unchanged by EMR.

**Figure 3:**
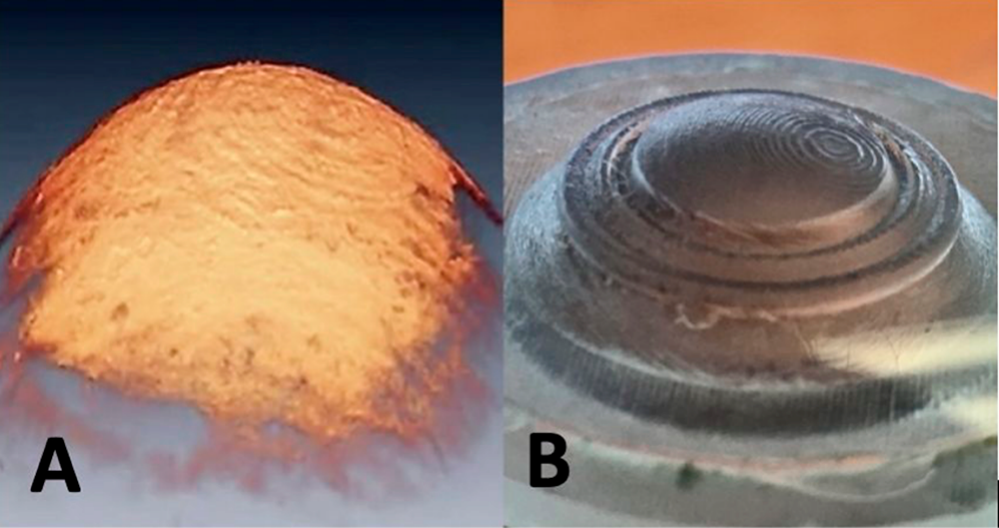
(A) SD-OCT constructed surface topography of treated cornea. (B) Close up of 3D-printed press mold used to fabricate the reshaping lens. The imprint of the layered rings resulting from the vertical resolution of our lithiographic printer are apparent on the corneal surface.

To further evaluate the surface contour of the treated cornea, a series of 2D SD-OCT sections were scanned, stacked, and processed to render a 3D topographical image, illustrated in Figure 3. The post-treatment corneal surface is uniform and qualitatively indistinguishable from an untreated specimen, with the exception of a series of shallow concentric circles inscribed onto its side. These circles originate from the 3D-printed mold used to fabricate the lens: the same features appear as vestiges of the 10-μm vertical resolution of our 3D printer. Although these ridges would need to be eliminated for preclinical *en vivo* studies, here they served as a convenient means to orient the cornea for subsequent imaging studies. Importantly, they also demonstrate that even subtle topographies of the reshaping lens appear to be readily transferred to the corneal surface during EMR.

To investigate the effect of EMR treatment on the molecular structure of the cornea, we employed second harmonic generation microscopy, SHG.^21,22^ SHG is a two-photon, nonlinear optical technique that has been used extensively to report on the underlying collagen organization of corneal tissues.^23,24^ For our purposes, the frequency-doubled SHG signal serves as a sensitive reporter of highly ordered noncentrosymmetric collagen bundles within the stroma: changing the spacing and/or lamellar organization of these bundles results in a loss of fluorescence signal. In order to provide an internal control for signal intensity, SHG images were collected on cornea treated with a “half-moon” reshaping lens, in which only half of the spherical reshaping surface was electrochemically active.

**Figure 4:**
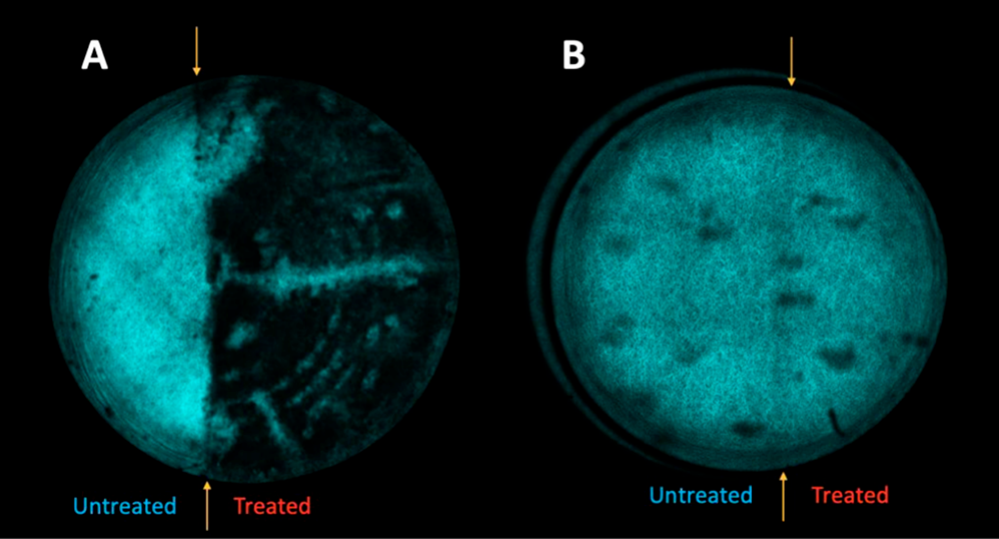
SHG tile scans of cornea treated with half-moon reshaping lenses. (A) Cornea subjected to 0.5 Hz anodic pulses at 1.5 V for a total electrochemical dose of 0.75 C. Note imprints of the concentric rings of the lens onto the treated, but not control, half of the cornea. (B) Cornea treated using looped double-potential-step chronocoulometry, with the applied potential alternating between 1.5 and −0.1 V at 0.5 Hz for a total anodic electrochemical dose of 0.75 C. The reshaping lens used in experiment B was polished to eliminate the underlying 10-μm vertical rings apparent in A. In each case, yellow arrows mark the boundary between the electrochemically inactive vs. electrochemically active halves of the reshaping lenses.

Figure 4A shows a post-therapy SHG tiled-scan image of a cornea treated with a 0.5 Hz pulsed potential at 1.5 V for 0.75 C, using a half-moon lens. Although the cornea remains visually transparent, SHG microscopy shows a clear loss in signal for the half of the tissue that was treated. One possible explanation for this finding is oxidative damage^25,26^ caused by the electrochemical production of hypochlorite at the platinum-lens surface. To test this possibility, we carried out an identical experiment, but instead submerged the eye in phosphate buffer containing no chloride; in this case, the post-treatment image (see the Supporting Information) revealed only a very modest loss in SHG signal, suggesting minimal disruption of the macromolecular structure.

**Figure 5:**
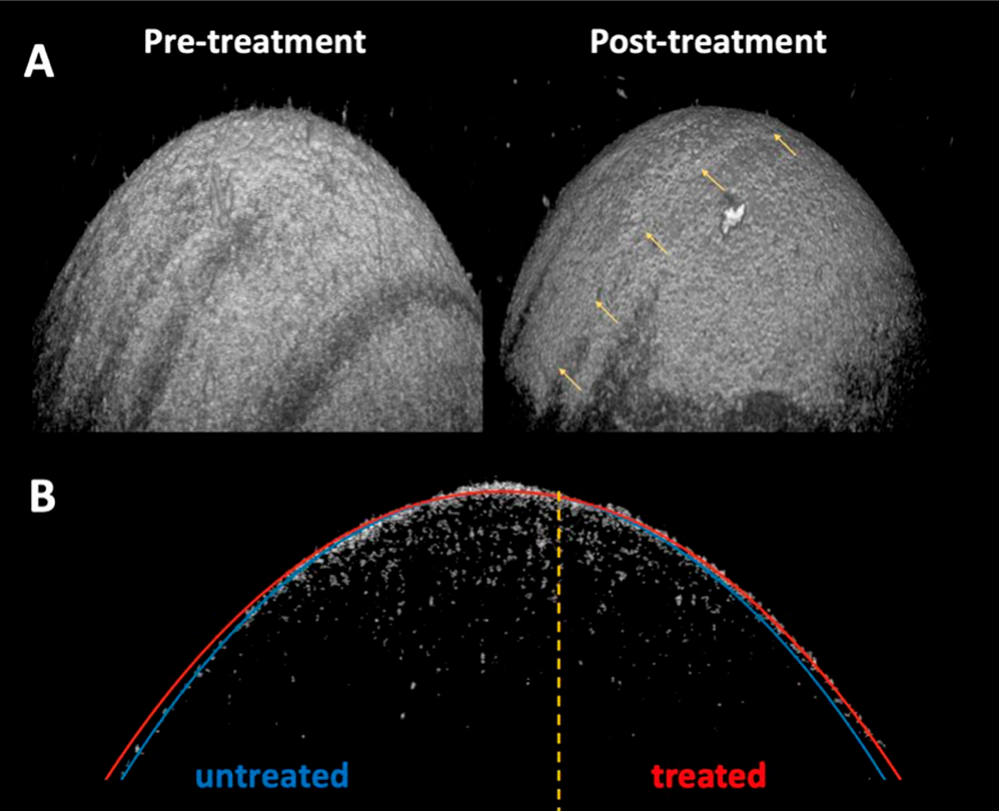
SD-OCT images of the cornea specimen shown in Figure 4B. Treatment protocol: looped double-potential-step pulse sequence between 1.5 and −0.1 V at 0.5 Hz for a net anodic charge of 0.75 C using a half-moon reshaping lens with a 7.35 mm focal length. (A) Topographical 3D image of the cornea pre- (left) and post-treatment (right). The yellow arrows on the image to the right highlight the boundary between the treated vs. untreated areas of the cornea. (B) A constructed 2D SD-OCT slice taken orthogonal to the treated/untreated boundary line at the corneal apex. The treated vs. untreated focal lengths were determined by fitting the respective half-spherical sections of the post-treatment cornea; the change in shape represents a −8.9 diopter change in refractive power: a 6.90 mm (untreated) vs. 7.35 mm (treated) radius of curvature.

In an attempt to limit damage from electrochemically generated ClO^–^ in the presence of chloride at physiological concentrations, we modified the treatment pulse sequence to include a cathodic cycle using looped double-potential-step chronocoulometry. In this method, the potential is repeatedly cycled between 1.5 V and −0.1 V at 0.5 Hz until the desired net anodic charge^27^ (i.e., the electrochemical treatment “dose”) is reached. This protocol was designed to electrochemically scavenge (during the cathodic step) any ClO^–^ produced during the anodic cycle of the treatment pulse sequence. Figure 4B shows the SHG image of a cornea treated using this strategy; topographical SD-OCT images of this same specimen pre- and post-treatment are shown in Figure 5, revealing shape change only of the treated hemisphere. Significantly, the SHG signal from the treated half of the tissue is indistinguishable from the control half, suggesting that therapy using this pulse sequence preserves the underlying macromolecular collagen structure of the stroma and may be an effective strategy to minimize damage from hypochlorite.

**Figure 6:**
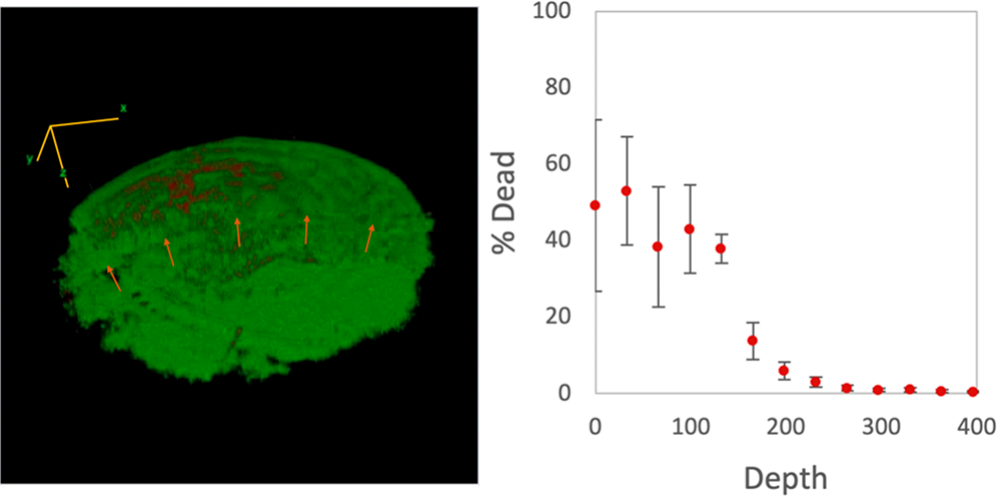
Left: Fluorescent confocal microscopy image of rabbit septal cartilage following a LIVE/DEAD cell-viability assay (Molecular Probes) performed on an eye specimen treated using a half-moon lens with a pulsed (1.5 V at 0.5 Hz; electrochemical dose of 0.15 C) waveform. Green dots correspond to living cells; red dots correspond to dead cells. Yellow arrows mark the boundary between the treated tissue (anterior) and the control region (posterior). Note: the stacked concentric rings apparent along the *z*-direction and spanning the entire corneal surface are an artifact of our data collection/processing procedure and do not appear in OCT images. Right: Plot of the percentage of dead cells as a function of depth through the cornea into the anterior chamber. Notably, virtually all of the dead cells are localized within the epithelium, with the stromal keratocytes intact. In all of the eyes treated using half-moon lenses with this treatment protocol (four), there were no statistically significant differences in the live/dead cell counts between the treated vs. control areas of the cornea.

Finally, to evaluate the effect of this pulsed EMR therapy on the viability of keratocytes within treated cornea, we employed confocal microscopy and a fluorescence live/dead assay, again using a half-moon reshaping lens to allow for an internal control on a single cornea specimen. After pulsed treatment, cornea were excised, stained, and mounted onto a custom 3D-printed microscope slide (see the Supporting Information) featuring a 3 mm aperture for light excitation. Vertically stacking fluorescence images (25 μm thick in the *z*-direction) obtained in the horizontal plane yields a three-dimensional rendering of the cells within the treated corneal volume. Figure 6 shows one such image, with green and red signifying live and dead cells, respectively. As with Figure 5, the boundary between the two halves of the treated cornea is readily apparent and is highlighted with arrows. Notably, as illustrated in the accompanying plot of the live/dead cell count as a function of corneal depth, there is remarkably little necrotic tissue, with virtually all of the cell injury localized to the epithelium.

## Conclusion

Pairing EMR with a customizable corneal reshaping contact lens offers the possibility of a molecular-based method to alter corneal curvature that does not require ablation of the native stromal tissue. Translation to clinical practice will require long-term viability studies on a live animal model, as well as comprehensive statistical analyses of the accuracy and range of possible refractive corrections achievable; whether treated eyes will display significant shape recidivism or maintain persistent recontouring is another important question to consider. That said, using *ex vivo* rabbit eyes, we have demonstrated here that it is possible to dial in corneal curvature over a wide range of refractive powers with no loss of optical transparency. Our current OCT imaging work, combined with SHG and confocal microscopy, establish that EMR enables extremely fine control of corneal contouring while maintaining the underlying macromolecular collagen structure and stromal cellular viability. Thus, not only is electrochemical corneal reshaping a potentially simple and ultralow-cost alternative for correcting routine refractive errors, it may also provide a viable modality for treating thin cornea and/or cornea that require large refractive corrections^28^—applications for which LASIK and other ablative techniques are not suitable.

## Supporting Information Available

To-scale engineering drawings of press mold EMR lens forms, lens mandrels, and mounting slides for corneal confocal microscopy; second-harmonic generation (SHG) microscopy images of a rabbit cornea treated by EMR therapy in chloride-free phosphate buffer (PDF)
