# Supplementary material for: Electromechanical Cornea Reshaping for Refractive Vision Therapy
Source: ACS Biomater Sci Eng. 2023 Jan 12;9(2):595–600. doi: 10.1021/acsbiomaterials.2c01177 (PMC9930080; doi:10.1021/acsbiomaterials.2c01177)
Supplement: Supplementary file 1 — ab2c01177_si_001.pdf [file ab2c01177_si_001.pdf]

Supporting information for:

Electromechanical Cornea Reshaping for Refractive Vision Therapy

Anna M. Stokolosa,[1] Jack Thomas-Colwell,[1] Katelyn K. Dilley,[2] Yueqiao Qu,[2,3] Charlotte Cullip,[1] Andrew E. Heidari,[2,3] Michelle Huang,[1] Nathalie Kerrigan,[1] Kellie Hsu,[1] Jack Leonard,[1] Karthik R. Prasad,[2] Brian J.F. Wong,[2,3,4*] Michael G. Hill[1*]

[1]Department of Chemistry, Occidental College, Los Angeles, CA USA

[2]Beckman Laser Institute & Medical Clinic, University of California, Irvine, Irvine, CA USA

[3]Department of Biomedical Engineering, University of California, Irvine, Irvine, CA USA

[4]Department of Otolaryngology-Head and Neck Surgery, University of California, Irvine, School of Medicine, Orange, CA USA

*To whom correspondence should be addressed.

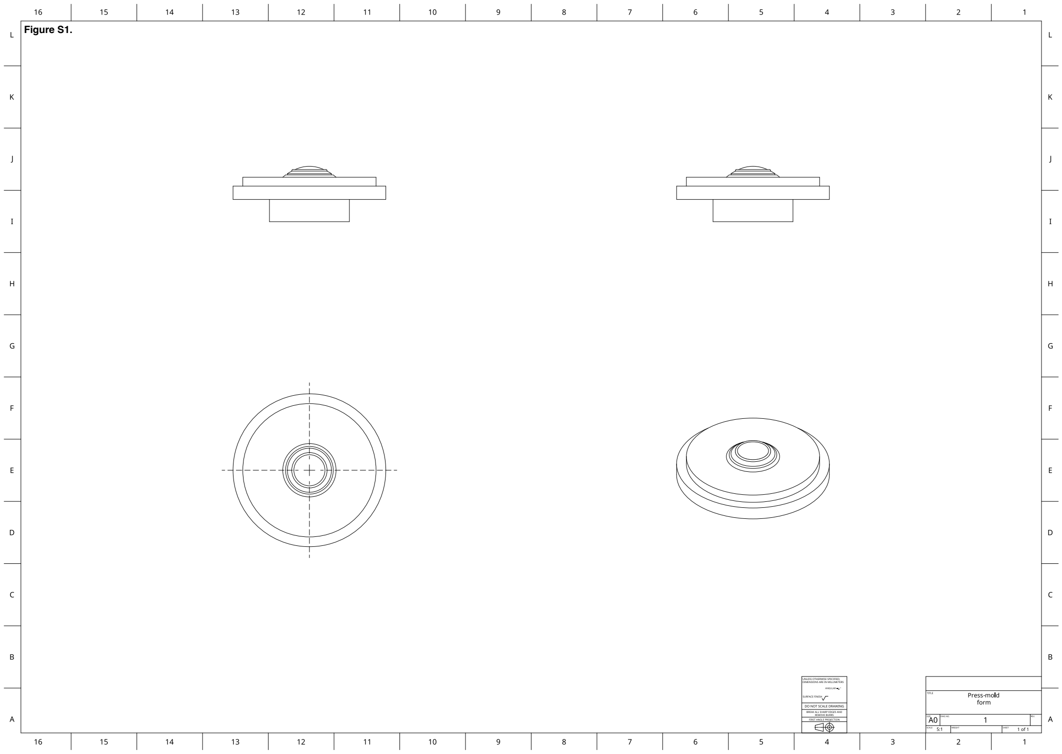

Figure S1. Half-scale drawing of 3D-printed form for press-molded reshaping lens, with a radius of curvature of 7.4 mm. All of the lenses used in this study used this same design with varying radii of curvature.

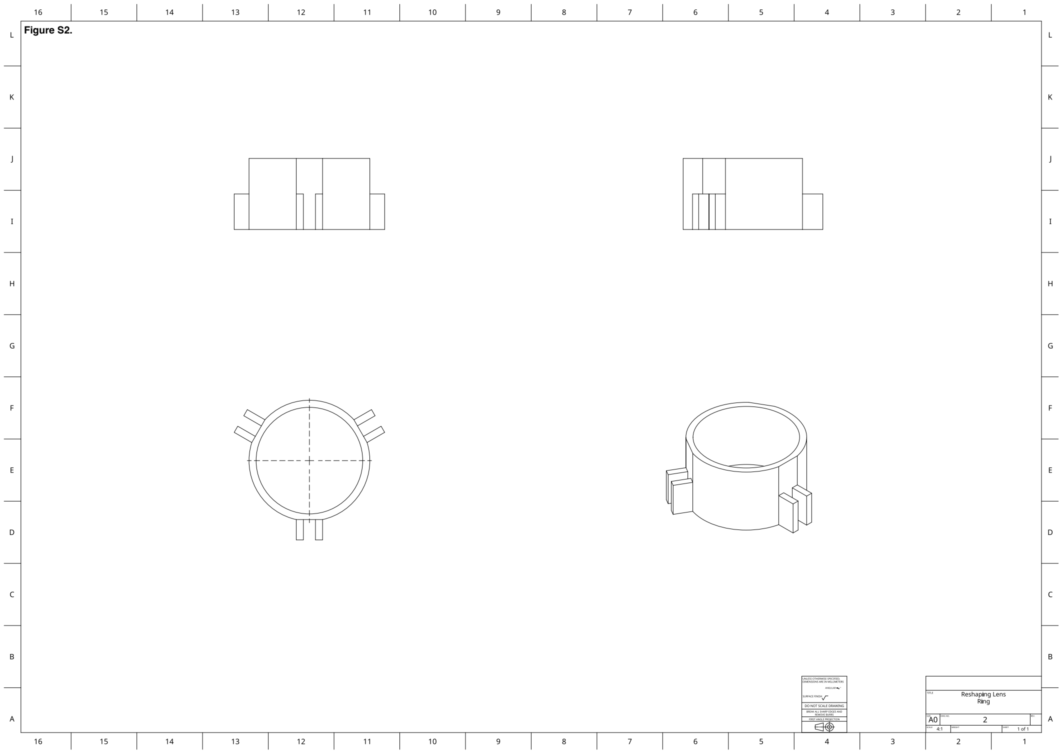

Figure S2. Half-scale drawing of 3D-printed ring for reshaping lens. All of the lenses used in this study used this same design.

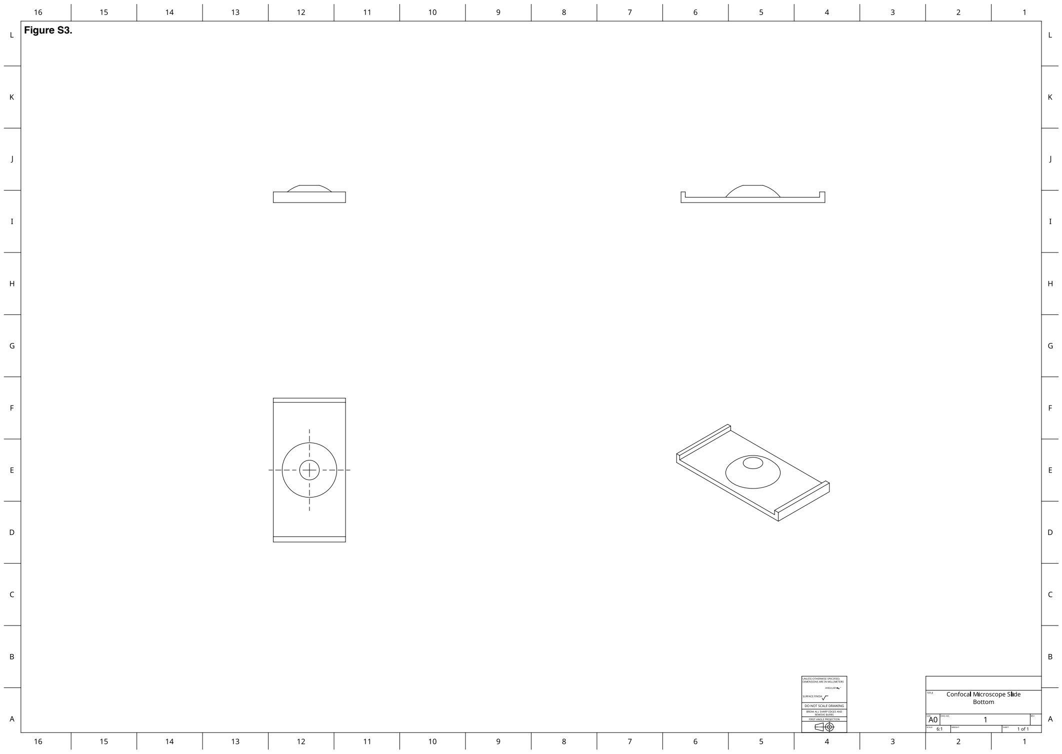

Figure S3. Half-scale drawing of 3D-printed mounting slide for confocal imaging. For imaging, the cornea was excised and mounted to the hemispherical feature of the slide and pressed between a corresponding cover slide (Figure S4).

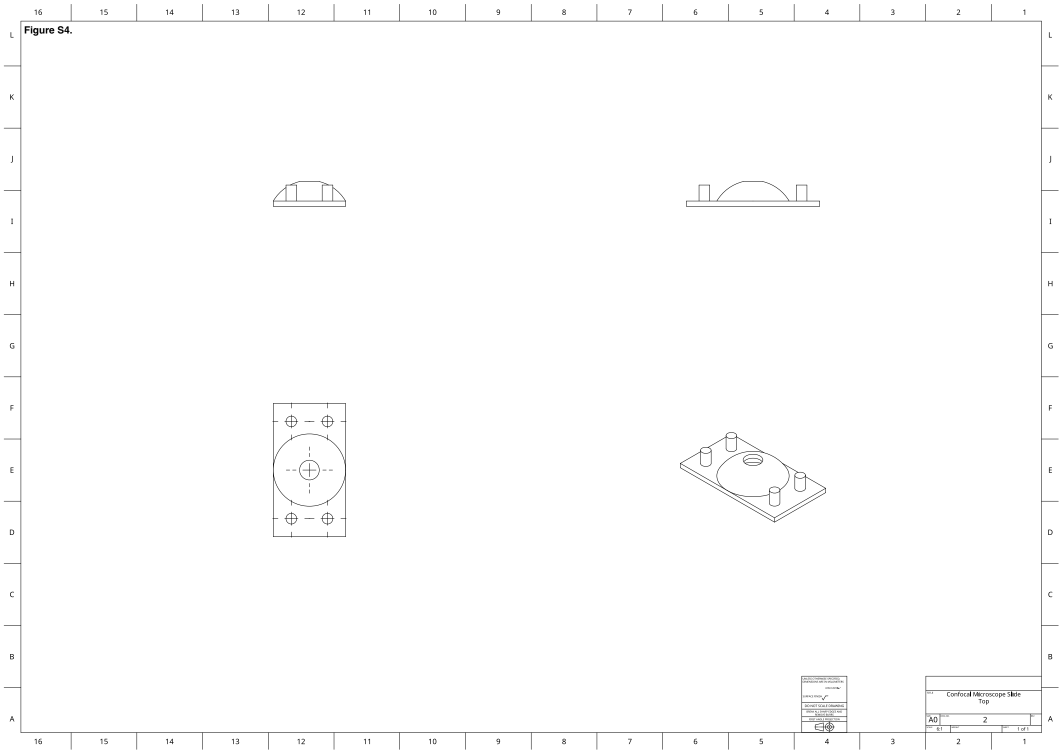

Figure S4. Half-scale drawing of top cover slide for confocal corneal imaging.

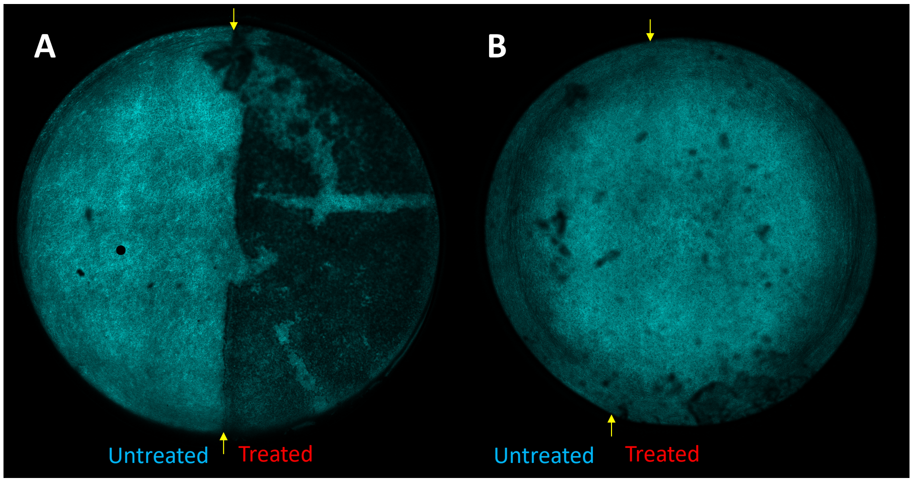

Figure S5. SHG tile scans of cornea treated with half-moon reshaping lens. (A) Cornea treated in (A) phosphate-buffered saline (PBS), and (B) chloride-free phosphate buffer, pH 7.4. Cornea were subject to 1Hz anodic pulses at 1.5 V vs. Ag pseudoreference for a total electrochemical dose of 56 mC. In each case, yellow arrows mark the boundary bewtween electrochemically inactive (untreated) vs. electrochemically active (treated) halves of the reshaping lens.
